# Persistence of intramyocardially transplanted murine induced pluripotent stem cell-derived cardiomyocytes from different developmental stages

**DOI:** 10.1186/s13287-020-02089-5

**Published:** 2021-01-08

**Authors:** Gabriel Peinkofer, Martina Maass, Kurt Pfannkuche, Agapios Sachinidis, Stephan Baldus, Jürgen Hescheler, Tomo Saric, Marcel Halbach

**Affiliations:** 1 Department of Internal Medicine III, University Hospital of Cologne, Cologne, Germany; 2 Center for Physiology and Pathophysiology, Institute of Neurophysiology, Medical Faculty, University of Cologne, Robert-Koch Str. 37, Cologne, 50931 Germany; 3 Marga-and-Walter-Boll Laboratory for Cardiac Tissue Engineering, University of Cologne, Cologne, Germany; 4 Department of Ophthalmology and Ocular GvHD Competence Center (P.S.), Medical Faculty, University of Cologne, Cologne, Germany; 5 Department of Pediatric Cardiology, University Hospital of Cologne, Cologne, Germany; 6 Center for Molecular Medicine, University of Cologne, Cologne, Germany

**Keywords:** Induced pluripotent stem cell-derived cardiomyocytes, Cell therapy, Cell persistence

## Abstract

**Background:**

Induced pluripotent stem cell-derived cardiomyocytes (iPSC-CM) are regarded as promising cell type for cardiac cell replacement therapy, but it is not known whether the developmental stage influences their persistence and functional integration in the host tissue, which are crucial for a long-term therapeutic benefit. To investigate this, we first tested the cell adhesion capability of murine iPSC-CM in vitro at three different time points during the differentiation process and then examined cell persistence and quality of electrical integration in the infarcted myocardium in vivo.

**Methods:**

To test cell adhesion capabilities in vitro, iPSC-CM were seeded on fibronectin-coated cell culture dishes and decellularized ventricular extracellular matrix (ECM) scaffolds. After fixed periods of time, stably attached cells were quantified. For in vivo experiments, murine iPSC-CM expressing enhanced green fluorescent protein was injected into infarcted hearts of adult mice. After 6–7 days, viable ventricular tissue slices were prepared to enable action potential (AP) recordings in transplanted iPSC-CM and surrounding host cardiomyocytes. Afterwards, slices were lysed, and genomic DNA was prepared, which was then used for quantitative real-time PCR to evaluate grafted iPSC-CM count.

**Results:**

The in vitro results indicated differences in cell adhesion capabilities between day 14, day 16, and day 18 iPSC-CM with day 14 iPSC-CM showing the largest number of attached cells on ECM scaffolds. After intramyocardial injection, day 14 iPSC-CM showed a significant higher cell count compared to day 16 iPSC-CM. AP measurements revealed no significant difference in the quality of electrical integration and only minor differences in AP properties between d14 and d16 iPSC-CM.

**Conclusion:**

The results of the present study demonstrate that the developmental stage at the time of transplantation is crucial for the persistence of transplanted iPSC-CM. iPSC-CM at day 14 of differentiation showed the highest persistence after transplantation in vivo, which may be explained by a higher capability to adhere to the extracellular matrix*.*

**Supplementary Information:**

The online version contains supplementary material available at 10.1186/s13287-020-02089-5.

## Background

Ischemic heart disease belongs to the most frequent causes of death worldwide [1]. Despite advances in medical and interventional treatment resulting in an improved myocardial function and survival, there is still no treatment available to successfully regenerate the lost heart muscle. Pluripotent stem cell-derived cardiomyocytes are considered a promising future option for regeneration of damaged heart tissue. Induced pluripotent stem cells (iPSC) possess similar differentiation capabilities as embryonic stem cells [2, 3] and can be differentiated into derivatives of all three germ layers including cardiomyocytes (iPSC-CM) [4, 5]. Recent advances in cell culture techniques allow for the derivation of large numbers of highly purified iPSC-CM [6, 7]. Experiments in small and large animal models of myocardial infarction (MI) showed safe application and an improved heart function after transplantation of iPSC-CM [8–12]. As result of these preclinical studies, the first clinical trials using human iPSC-CM were recently approved by regulatory authorities [13]. However, the mechanisms behind cell homing into recipient cardiac tissue or cell loss are poorly understood and the poor cell persistence after transplantation remains an unsolved challenge.

Several studies demonstrated that the percentage of cells remaining in the host tissue after transplantation is very low and ranges approximately between 2 and 11%. In a study using intramyocardial injection technique in a rat model of MI, the number of neonatal rat cardiomyocytes detected after 3 weeks varied between 8.2 ± 3.6% in a low cell dose group (5 × 10^6^ cells, injected into five sites of the infarction border zone) and 9.6 ± 2.4% in a high cell dose group (25 × 10^6^ cells, injected into five sites of infarction border zone) [14]. Hou et al. tested different administration routes in an ischemic swine model. Intramyocardial injection of peripheral blood mononuclear cells showed highest cell persistence (11 ± 3%) in comparison to intracoronary (2.6 ± 0.3%) and interstitial retrograde coronary venous administration (3.2 ± 1%) 1 h after cell delivery [15]. The optimal delivery route of cells will also depend on clinical settings like planned open chest surgery or catheter interventions. After direct myocardial injection, cells can be lost immediately because of leakage through the injection channel [16], and after intramyocardial and intracoronary injection, cells can be washed away through the circulation [15, 17]. Besides immediate cell loss after transplantation also ischemia and apoptotic cell death decrease cell retention [18, 19]. Co-transplantation of different cell types like smooth muscle cells, fibroblasts or MSCs may influence cell retention. Co-transplantation of embryonic stem cell-derived cardiomyocytes (ESCM) with mesenchymal stem cells (MSC) combined in in vitro grown micro tissue increased cell retention more than tenfold within the first days after transplantation [20].

Among other factors, the developmental stage of transplanted cells has a major effect on their persistence and integration. Adult cardiomyocytes do not survive transplantation [19], while juvenile native cardiomyocytes and immature iPSC-CM survive and integrate functionally [19, 21, 22]. Even during early development, cardiomyocytes undergo substantial morphological and physiological changes [23–25], which influence their fate after transplantation. Fetal murine cardiomyocytes at an intermediate stage (day 14.5 post coitum) have the highest persistence and quality of electrical integration as compared to earlier (day 12.5 post coitum) and later stages (day 18.5 post coitum) [26]. iPSC-CM undergo similar developmental changes in vitro as native cardiomyocytes in vivo [27], making it likely that the fate of transplanted cells will by significantly affected by their specific developmental stage.

Therefore, in the present study, we analyzed adhesion of murine iPSC-CM from different stages of differentiation (days 14, 16, and 18) to fibronectin-coated culture dishes and native cardiac extra cellular matrix (ECM) scaffolds in vitro and cell persistence and quality of electrical integration after transplantation into infarcted mouse heart in vivo*.* Our data demonstrate that day 14 iPSC-CM exhibit the highest adhesion ability in vitro and the highest survival rate with good functional integration potential in vivo. These results shed new light on stage-dependent iPSC-CM persistence and integration.

## Methods

### iPS cell culture and cardiac differentiation

The murine iPS cell line TiB7.4 was used, which was generated from murine tail tip fibroblasts isolated from 129S4/Sv4JaeJ × C57Bl/6 mice [28]. Cells were transfected with the α-PIG plasmid vector containing the PAC-(encoding puromycin *N*-acetyl-transferase) and IRES (*internal ribosomal entry site*) flanked eGFP-gene under control of the α-Myosin Heavy Chain promoter (GenBank Accession No. U71441), as described previously [22, 29]. iPS cells were grown on inactivated murine embryonic fibroblasts in DMEM supplemented with 15% fetal calf serum, 1× non-essential amino acids, 2 mM l-glutamine, 100 μM ß-mercaptoethanol (all reagents were purchased from Thermo Fisher Scientific, Waltham, USA), and 1000 IU/ml leukemia-inhibiting factor (Merck Millipore, Billerica, USA). Murine embryonic fibroblasts were prepared from transgenic C57BL6 mice carrying a neomycin resistance gene at embryonic day 14.5 and inactivated by mitomycin C treatment. iPS cells were passaged every second or third day; cells were trypsinized and 0.5 × 10^5^ cells were added to a 6-cm dish with preplated murine embryonic fibroblasts (0.8 × 10^5^/dish).

For cardiac differentiation, 1 × 10^6^ iPS cells were suspended in 10 cm bacterial dishes in 14 ml Iscove’s modified Dulbecco’s medium (IMDM) supplemented with 20% fetal calf serum, 100 μM ß-mercaptoethanol, 1× non-essential amino acids, and 50 μg/ml ascorbic acid (Thermo Fisher Scientific) and placed on a horizontal shaker for 2 days to allow embryoid body formation. After 2 days, 30,000 embryoid bodies were transferred to a spinner flask filled with 200 ml of IMDM differentiation medium. Starting at day 9, 8 μg/ml puromycin (InvivoGen Europe, Toulouse, France) was added for cardiomyocyte purification, and medium with fresh puromycin was changed every second day. At day 14, day 16, or day 18, purified iPSC-CM clusters were dissociated into single cardiomyocytes with 0.25% trypsin-EDTA (Thermo Fisher Scientific) supplemented with 5 U/ml of DNAse I (Sigma-Aldrich, Taufkirchen, Germany) for further use.

### Quantification of iPSC-CM adhesion in vitro

At day 14, day 16, and day 18 of cardiac differentiation, dissociated iPSC-CM were plated on fibronectin-coated (20 μg/ml for 2 h at 37 °C; Sigma-Aldrich) 3-cm dishes. In each approach, two drops containing 20,000 cells in 100 μL cell culture medium each were added to separate fibronectin-coated cell culture dishes. One drop was incubated for 5 min and the other for 10 min. Afterwards, the dishes were washed with 1.5 ml PBS^−/−^ for 1 min. Cells attached to the surface were counted using a microscope (Axiovert 200, Zeiss, Oberkochen, Germany) and the ImageJ program [30].

### Preparation of ventricular slices

Ventricular slices were prepared as described before [31, 32]. The hearts were resected and perfused with ice-cold Tyrode solution (composition in mmol/l: NaCl 136, KCl 5.4, NaH_2_PO_4_ 0.33, MgCl_2_ 1, glucose 10, Hepes 5, 2,3-butanedione monoxime 30; pH 7.4 adjusted with NaOH; all chemicals were purchased from Sigma Aldrich). Ventricles were separated from the atria and embedded in 4% low melt agarose (Roth, Karlsruhe, Germany). Short axis slices (150 μm) were cut with a microtome (Leica VT1000S; Leica Microsystems, Wetzlar, Germany) and stored in Tyrode solution containing 0.9 mmol/L Ca^2+^ for 30 min on ice. Afterwards, slices were either frozen for decellularization or transferred to DMEM at 37 °C aerated with 95% O_2_ and 5% CO_2_ and allowed to recover for another 30 min before action potential (AP) recordings.

### Decellularization of ventricular slices

Ventricular heart slices were frozen at − 20 °C for at least 24 h. To remove all cellular components from the ECM structure, slices were first rinsed with PBS^−/−^ at 37 °C for 15 min. Afterwards, treatment was continued with 1% SDS (SDS, Sigma Aldrich) in PBS^−/−^ for 2 h at 37 °C. SDS was washed out with PBS^−/−^ for 15 min at 37 °C. Subsequently, slices were rinsed with 3% Triton x-100 (3% Triton X-100, Sigma Aldrich) in PBS^−/−^ for 2 h at 37 °C. Finally, Triton X-100 was washed out with PBS^−/−^ for 30 min at 37 °C. Decellularized ECM scaffolds were used immediately or stored in PBS^−/−^ at 4 °C.

### Recellularization of ECM scaffolds

**Fig. 1:**
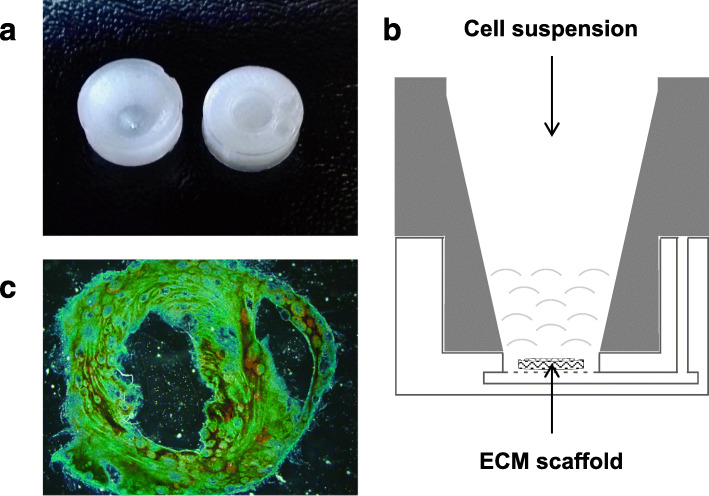
Decellularized ECM scaffold on a funnel dish. **a** Representative picture of a custom-made funnel dish, upper part left, and lower part right. **b** Schematic view of a funnel dish. **c** Decellularized ECM scaffold stained with Masson-Trichrome-Staining. Green: collagen, Red: keratin and muscle fibers, Pink: cytoplasm

ECM scaffolds were carefully placed in a custom made “funnel dish” (Fig. 1). Funnel dishes were printed using Ultimaker2 3D printer (Ultimaker, Utrecht, Netherlands). Subsequently, 100 μl of cell suspension containing 2.0 × 10^5^ day 14, day 16, or day 18 iPSC-CM in iPSC-CM cell culture medium were added. Funnel dishes were incubated at 37 °C with 5% CO_2_ for 1 h. Scaffolds were rinsed with iPSC-CM cell culture medium for 5 min and incubated in iPSC-CM cell culture medium at 37 °C and 5% CO_2_ for 7 days. Medium was changed every second day. Afterwards, genomic DNA from recellularized ECM scaffolds was isolated for cell quantification using qPCR.

### DNA isolation and quantitative PCR analysis

To quantify cell persistence and adhesion, genomic DNA from the whole hearts, dissociated iPSC-CM and recellularized ECM scaffolds were isolated using Qiagen DNeasy Blood & Tissue kit (Qiagen, Hilden, Germany). Quantitative real-time PCR was performed as described before [14, 33]. In short, the primer against the PAC-gene (Custom TaqMan® Gene Expression Assay MousePURO, Applied Biosystems, Life Technologies Corporation, Carlsbad, USA; Sequence for product design: ATGACCGAGTACAAGCCCACGGTGCGCCTCGCCACCCGCGACGACGTCCCCAGGGCCGTACGCACCCTCGCCGCCGCGTTCGCCGACTACCCCGCCACGCGCCACACCGTCGATCCGGACCGCCACATCGAGCGGGTCACCGAGCTGCAAGAACTCTTCCTCACGCGCGTCGGGCTCGACATCGGCAAGGTGTGGGTCGCGGACGACGGCGCCGCGGTGGCGGTCTGGACCACGCCGGAGAGCGTCGAAGCGGGGGCGGTGTTCGCCGAGATCGGCCCGCGCATGGCCGAGTTGAGCGGTTCCCGGCTGGCCGCGCAGCAACAGATGGAAGGCCTCCTGGCGCCGCACCGGCCCAAGGAGCCCGCGTGGTTCCTGGCCACCGTCGGCGTCTCGCCCGACCACCAGGGCAAGGGTCTGGGCAGCGCCGTCGTGCTCCCCGGAGTGGAGGCGGCCGAGCGCGCCGGGGTGCCCGCCTTCCTGGAGACCTCCGCGCCCCGCAACCTCCCCTTCTACGAGCGGCTCGGCTTCACCGTCACCGCCGACGTCGAGGTGCCCGAAGGACCGCGCACCTGGTGCATGACCCGCAAGCCCGGTGCCTGA) was used to detect iPSC-CM. As housekeeping gene, Gene Expression Assay MouseTuba4a (Mm00849767_s1, Applied Biosystems) was used. Samples with known numbers of PAC-positive iPSC-CM and PAC-negative native ventricular cardiomyocytes were used to derive a calibration curve for the calculation of PAC–positive/total DNA ratio in recipient hearts. For PAC-positive iPSC-CM detection on ECM scaffolds, iPSC-CM serial dilution was used to generate a calibration curve for the calculation of absolute cell numbers of remaining PAC-positive cells.

### Microarray gene expression analysis

Microarray analysis was done at the transcriptomics core facility at the Center for Molecular Medicine Cologne (CMMC), as described previously [34, 35]. Briefly, total RNA for global gene expression analysis was extracted from dissociated day 14, day 16, and day 18 iPSC-CM using RNeasy mini kit (Qiagen, Hilden, Germany). For each analysis, triplicates made of different cell batches were used. For microarray labeling, 12.5 μg amplified RNA was hybridized on Mouse Genome 430 version 2.0 arrays (Affymetrix, Santa Clara, CA, USA). After staining (Affymetrix Fluidics Station-450), arrays were scanned with Affymetrix Gene-Chip Scanner-3000-7G, while quality control matrices were confirmed with Affymetrix GCOS software. Statistical data analysis was performed using the Transcriptome Analysis Console (TAC) software from Applied Biosystems (Applied Biosystems, Foster City, CA, USA). Comparison was performed by the ANOVA method (ebayes) to generate the differentially regulated transcripts with at least a 2-fold change (*p* value < 0.05). For more details, please see supplemental methods.

### iPSC-CM transplantation

At day 14 and day 16 of cardiac differentiation, iPSC-CM were transplanted into the infarcted hearts of syngenic female 129S4/Sv4JaeJ × C57Bl/6 mice (> 8 weeks) as described before [36]. After permanent LAD ligation, dissociated iPSC-CM were injected with a Hamilton syringe (H. Faust GmbH, Rheinbach, Germany) attached to a 29-gauge needle (Sigma-Aldrich) into two sites (500,000 cells/10 μl 0.9% NaCl solution at each site) of the infarct border zone. For further analysis, animals were kept alive for 6–7 days after surgery. All experiments conformed to the guidelines of the local animal welfare committee and to the Directive 2010/63/EU of the European Parliament.

### AP recordings

Intracellular AP recordings in ventricular slices were performed with sharp glass microelectrodes (15–40 MΩ when filled with 3 mol/l KCl; World Precision Instrument, Sarasota, USA) as described before [22, 37]. eGFP-positive iPSC-CM could be identified by their green fluorescence, enabling a precise positioning of the recording electrode in graft or host tissue. A defined beating frequency was applied with a SD9 square pulse stimulator (Grass Technologies, West Warwick, USA) using a unipolar custom-made stimulation electrode. Signals were amplified with a SEC-10LX amplifier (npi electronic, Tamm, Germany) and acquired with the Pulse software (HEKA, Lambrecht/Pfalz, Germany). Data were analyzed offline with Mini Analysis (Synaptosoft, Fort Lee, USA). Because electrical excitation originated from host tissue, we determined the temporal interdependency of stimulation artifacts and APs recorded intracellularly in transplanted cardiomyocytes as indicator of an electrical integration. The quality of electrical integration could be assessed by the maximal stimulation frequency without conduction blocks, i.e., the maximal stimulation frequency leading to a 1:1 generation of APs after every stimulus.

### Statistics

All data are presented as mean ± S.E.M. Two groups of data were tested for statistical significance by Student’s *t* test or, if normality test failed, by Mann–Whitney rank sum test. More than two groups were tested by one-way ANOVA with post-test or, if normality test failed, by one-way ANOVA on ranks with post-test. A two-sided *p* value < 0.05 was considered statistically significant. SigmaStat (Systat, Erkrath, Germany), GraphPad Prism 8.0 (GraphPad Software, San Diego, USA), and SPSS Statistics version 23 (IBM, Armok, NY, USA) were used for all calculations.

## Results

### Adhesion of iPSC-CM to fibronectin-coated dishes

**Fig. 2:**
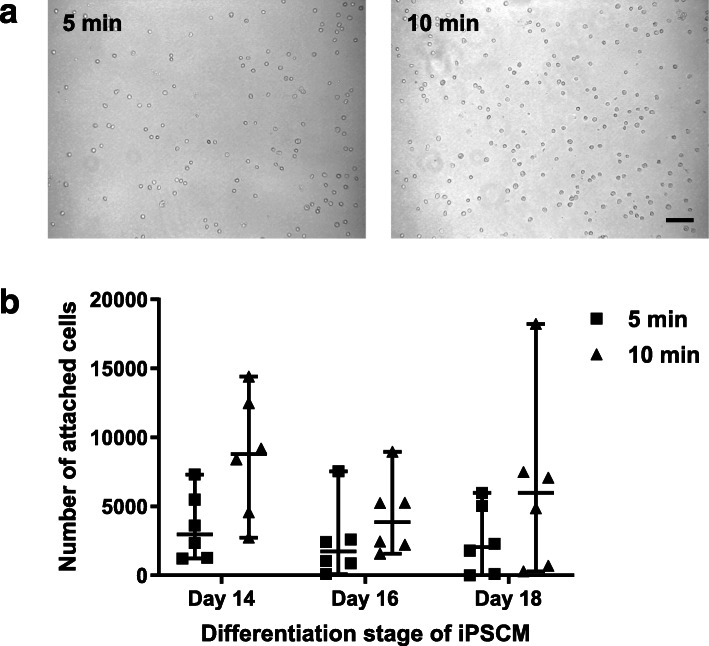
Adhesion of iPSC-CM to fibronectin-coated dishes. **a** Representative pictures of attached iPSC-CM 5 min (left panel) and 10 min (right panel) after plating on fibronectin-coated cell culture dishes. Scale bar 100 μm. **b** Number of attached iPSC-CM plated at different stages (14 days, 16 days, and 18 days after starting the differentiation process). 20,000 cells were plated in 100 μl drops per dish. Cells were photographed and counted 5 (square) or 10 (triangle) minutes after plating. All data are presented as mean + S.E.M. Statistical analysis was performed using one-way ANOVA with post-test, 5 min: *p* = 0.709; 10 min *p* = 0.326

To test differences in adhesion capability of iPSC-CM at day 14, day 16, and day 18 of differentiation, which might contribute to differences in persistence after transplantation, their attachment to fibronectin-coated dishes was assessed in vitro. After 5 min of cultivation, 3542 ± 999 cells at day 14, 2430 ± 1093 cells at day 16 and 2532 ± 1017 cells at day 18 of differentiation remained attached to the surface of the fibronectin-coated dishes. After 10 min of cultivation, 8634 ± 1824 cells at day 14, 4288 ± 1134 cells at day 16, and 6442 ± 2668 cells at day 18 of differentiation were attached (Fig. 2). The differences between the three groups were not statistically significant (5 min, *p* = 0.709; 10 min, *p* = 0.326).

Cell diameter of attached round shaped iPSC-CM was similar at all time points. Day 14 iPSC-CM showed an average diameter of 65 ± 2 μm, day 16 iPSC-CM of 68 ± 2 μm, and day 18 iPSC-CM of 65 ± 2 μm (*n* = 53, *p* = 0.285).

### Adhesion of iPSC-CM to ECM scaffolds

**Fig. 3:**
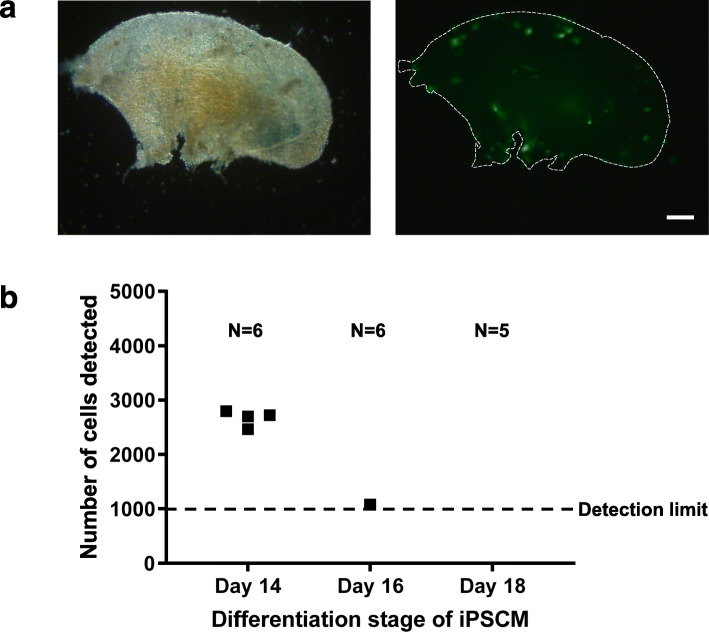
Adhesion of iPSC-CM to ventricular ECM scaffolds. **a** Representative picture of decellularized ventricular heart tissue (left) with attached eGFP-positive iPSC-CM (right). Scale bar 100 μm. **b** Absolute number of iPSC-CM detected by qPCR. iPSC-CM were added to ECM scaffolds at different time points after starting the differentiation process (14, 16, and 18 days). The number of attached cells was below the detection limit of the qPCR in 2/6 samples for iPSC-CM at day 14, 5/6 samples for iPSC-CM at day 16 and all 5 samples for iPSC-CM at day 18 (data points not included in the diagram)

To further investigate the adhesion capability of iPSC-CM from different stages (day 14, day 16, and day 18), attachment to ECM scaffolds gained from decellularized ventricular slices was tested. Six samples of iPSC-CM at day 14 of differentiation were analyzed. In two samples, the number of attached cells was below the detection limit of the qPCR method (i.e., below 1000 cells). In the other four samples in average 2671 ± 71 iPSC-CM per slice could be detected after 7 days of cultivation on ECM scaffolds. When iPSC-CM at day 16 of differentiation were examined, the number of attached cells was below the detection limit in five out of six samples. In the remaining sample, 1076 iPSC-CM were detected. The number of attached iPSC-CM at day 18 of differentiation was below the detection limit in all samples (*n* = 5) (Fig. 3).

### Persistence of transplanted iPSC-CM

**Fig. 4:**
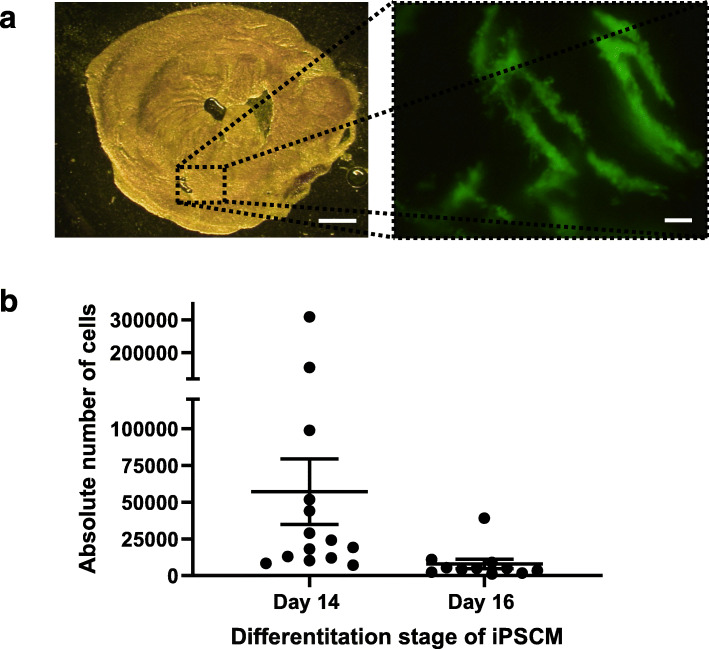
Cell persistence after transplantation of iPSC-CM in infarcted mouse hearts. **a** Representative image of eGFP-positive iPSC-CM within a ventricular slice of the recipient heart, 7 days after transplantation. Left scale bar 1 mm, right scale bar 100 μm. **b** Number of iPSC-CM assessed by qPCR 6–7 days after injecting 2 × 500,000 iPSC-CM in the infarct border zone. iPSC-CM were transplanted at 14 or 16 days after starting the differentiation process. Mean persistence was approximately 7-fold higher in cells transplanted at day 14 of differentiation compared to day 16 iPSC-CM. Statistical analysis was performed using the Mann–Whitney rank sum test, *p* < 0.001

To test cell persistence after transplantation of iPSC-CM at different developmental stages, day 14 and day 16 iPSC-CM were transplanted into the infarct border zone of *N* = 14 and *N* = 11 recipient mice, respectively. Day 18 iPSC-CM were not included in these in vivo experiments due to the very low attachment to ECM scaffolds observed in vitro. Areas of green fluorescent transplanted cells were clearly visible by fluorescence microscopy, revealing the survival of iPSC-CM at 7 days after transplantation (Fig. 4a). Quantification of persistence by qPCR revealed that iPSC-CM transplanted at day 14 of differentiation had an approximately 7-fold higher persistence in host tissue than iPSC-CM transplanted at day 16 of differentiation (Fig. 4b), with an average of 57,173 ± 8343 remaining day 14 iPSC-CM per host ventricle and 7983 ± 3241 remaining day 16 iPSC-CM (*p* < 0.001, Fig. 4b). This corresponded to a mean persistence of 5.7% of transplanted day 14 iPSC-CM and 0.8% of day 16 iPSC-CM. A maximal number of 308,713 (30.9%) and a minimal number of 7244 cells (0.7%) were detected after transplantation of day 14 iPSC-CM. For day 16 iPSC-CM, the maximal number was 39,135 (3.9%) that was observed in only one animal and the minimal number 1183 iPSC-CM (0.11%).

### Functional integration of transplanted iPSC-CM

**Fig. 5:**
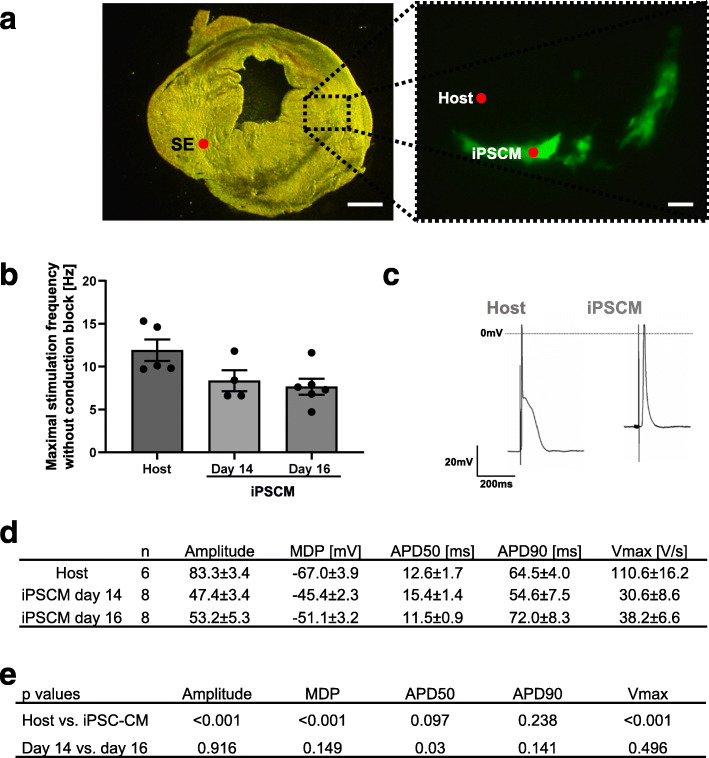
Functional integration of transplanted iPSC-CM into the myocardium. **a** Representative bright field image of a ventricular heart slice (left picture) with retained eGFP-positive iPSC-CM (right picture) 7 days after injection of 2 × 0.5 × 10^6^ day 16 iPSC-CM. Left scale bar 1 mm, right scale bar 100 μm. The positions of the stimulation electrode (SE) and the recordings in host tissue and iPSC-CM are marked. **b** The quality of electrical integration of day 14 iPSC-CM, day 16 iPSC-CM, and native host cardiomyocytes assessed by the maximal stimulation frequency without conduction block. **c** Representative action potentials of a host cardiomyocyte and transplanted iPSC-CM. **d** Action potential properties of transplanted day 14 iPSC-CM (*n* = 6), day 16 iPSC-CM (*n* = 8), and host cardiomyocytes (*n* = 8). MDP maximal diastolic potential, *V*max maximal upstroke velocity, APD50 action potential duration at 50% of repolarization, APD90 action potential duration at 90% of repolarization. **e** Statistical analyses of action potential properties. Statistical analysis were performed using one-way ANOVA with post-test for host vs. iPSC-CM and Student’s *t* test for day 14 vs. day 16 iPSC-CM

Sharp electrode action potential measurements in healthy heart tissue as well as in green fluorescent iPSC-CM (Fig. 5a) were performed to evaluate the quality of electrical integration of transplanted iPSC-CM into the recipient myocardium and to analyze electrophysiological properties of transplanted day 14 (*N* = 6) and day 16 (*N* = 8) iPSC-CM in comparison to host (*N* = 8) cardiomyocytes.

The maximal stimulation frequency without conduction block, indicating the quality of integration of transplanted cells, was not significantly different between the two transplanted groups and was 8.3 ± 1.2 Hz for day 14 iPSC-CM and 7.6 ± 0.9 Hz for day 16 iPSC-CM (*p* = 0.653). The maximal stimulation frequency without conduction block in the surrounding native heart tissue was significantly higher than in both iPSC-CM groups (11.9 ± 1.2 Hz, *p* = 0.038, Fig. 5b).

Analyses of action potential properties showed significant differences between host tissue and iPSC-CM (Fig. 5c) in amplitude (host tissue 83.3 ± 3.4 mV, day 14 iPSCM 47.4 ± 3.4 mV, day 16 iPSCM 53.2 ± 3.6 mV, *p* < 0.001), maximal diastolic potential (MDP; host tissue − 67.0 ± 3.9 mV, day 14 iPSCM − 45.4 ± 2.3 mV, day 16 iPSCM − 51.1 ± 3.2 mV, *p* < 0.001) and upstroke velocity (*V*_max_; host tissue 110.6 ± 16.2 mV, day 14 iPSCM 30.6 ± 8.6 mV, day 16 iPSCM 38.2 ± 6.6 mV, *p* < 0.001; Fig. 5d, e). No significant differences were found between host tissue and iPSCM in action potential duration at 50% of repolarization (APD50; host tissue 12.6 ± 1.7 ms, day 14 iPSCM 15.4 ± 1.4 ms, day 16 iPSCM 11.5 ± 0.9 ms, *p* = 0.097) and 90% of repolarization (APD90; host tissue 64.5 ± 4.0 ms, day 14 iPSCM 54.6 ± 7.5 ms, day 16 iPSCM 72.0 ± 8.3 ms, *p* = 0.238; Fig. 5d, e).

Comparison of action potential properties between transplanted day 14 and day 16 iPSC-CM revealed no significant differences between the two groups in amplitude (day 14 iPSCM 47.4 ± 3.4 mV, day 16 iPSCM 53.2 ± 5.3 mV, *p* = 0.916), MDP (day 14 iPSCM − 45.4 ± 2.3 mV, day 16 iPSCM − 51.1 ± 3.0 mV, *p* = 0.149), APD90 (day 14 iPSCM 54.6 ± 7.5 ms, day 16 iPSCM 72.0 ± 8.3 ms, *p* = 0.141), and upstroke velocity (*V*_max_; day 14 iPSCM 30.6 ± 8.6 V/s, day 16 iPSCM 38.2 ± 6.6 V/s, *p* = 0.496; Fig. 5d, e). However, a significant difference was found in APD50 (day 14 iPSCM 15.4 ± 1.4 ms, day 16 iPSCM 11.5 ± 0.9 ms, *p* = 0.03; Fig. 5d, e).

### Gene expression analysis

Most common cardiac integrin subunits α1 (Itga1), α5 (Itga5), α7 (Itga7), β1 (Itgb1), and β8 (Itgb8) and integrin-binding protein fibulin 1(Fbln1), fibroblast growth factor 1 (FGF1), and myosin heavy poloypeptide 9 (Myh9) have been analyzed. Integrin subunit genes Itga5, Itga7, and Itgb8 and integrin-binding protein genes Fbln1 and FGF1 showed lower expression levels (at least a 2-fold change with *p* values < 0.05) in day 14 iPSC-CM than in day 18 iPSC-CM. Only Myh9 was expressed higher in day 14 iPSC-CM than in day 18 iPSC-CM. No differences in gene expression level (< 2-fold change and/or *p* > 0.05) were detected for Intga1, Intga5, and Intgb1 as well as Fbln1 (supplemental Fig. 1 and supplemental Tables 1 and 2).

Gene expression profiles in day 14, day 16, and day 18 iPSC-CM of cardiac gap junction proteins α1 (Gja1; Connexin 43), α5 (Gja5; Connexin 40), and γ1 (Gjc1; Connexin 45) were also analyzed. Gja1 and Gjc1 showed no difference (< 2-fold change and/or *p* > 0.05) in day 14, day 16, and day 18 iPSC-CM. Gja5 showed slightly lower expression in day 14 than in day 16 (− 2.14 fold change, *p* = 0.005) and day 18 (− 2.23 fold change, *p* = 0.002) iPSC-CM (supplemental Fig. 2 and supplemental Tables 3 and 4).

## Discussion

Cardiac cell therapy is regarded as a promising approach to regenerate lost myocardium and restore cardiac function in heart failure. iPSC-CM represent a suitable cell type for a true exogenous cell replacement, since they have functional and structural properties like native cardiomyocytes [4, 5], can be produced in large numbers [6, 7], and reduce immune rejection which would occur with allogeneic cells [38]. Persistence and survival of transplanted cardiomyocytes are of major importance for replacement of lost myocardium, but were reported to be low in previous studies [14, 15, 33]. In the present work, we demonstrate for the first time that the persistence of transplanted iPSC-CM strongly depends on their developmental stage at the time of transplantation and that differences in the capability of iPSC-CM to attach to ECM in vitro correlate with their short-term survival rate in vivo.

It is well known that the developmental stage at the time of transplantation is crucial for the persistence and survival of transplanted cells, since dissociated adult cardiomyocytes do not survive transplantation at all [19], while immature cardiomyocytes of different developmental stages have been shown to integrate into the host tissue in many studies [14, 21, 22, 26]. But even during maturation, properties of cardiomyocytes undergo substantial changes [23–25, 27] that might influence their fate after transplantation. First evidence of differences in the persistence of immature cardiomyocytes transplanted at different developmental stages was provided by a study that compared fetal cardiomyocytes at day 9.5, day 14.5, and day 18.5 after injection into mouse hearts [26]. The intermediate stage was most efficient regarding cell persistence and functional integration. Six days after transplantation, the number of transplanted cells was almost 4-fold higher in the intermediate stage than in the late stage and more than 6-fold higher than in the early stage, i.e., the magnitude of the influence of the developmental stage on persistence was comparable to that found in the present study. Day 14.5 of fetal development and day 14 of iPSC-CM differentiation provided the best persistence, which is surprising taking into account the differences between the development of native cardiac tissue and iPSC-CM described before [27], showing that native cardiomyocytes at embryonic days 12–14 have similar electrophysiological characteristics as day 18 murine iPSC-CM in vitro.

Several mechanisms underlie loss of injected cells, including apoptosis, necrosis, and washout from the injection site [16, 18, 19]. Several studies in large and small animals revealed that more than 90% of transplanted cells are washed out early after injection [39–41]. This may be linked to the capability of cells to attach to the ECM. Furthermore, lack of interaction between ECM and transplanted cells might induce anoikis [42], which might be prevented by cell adhesion. Cardiomyocytes seem to be very selective for attachment to different substrates in vitro. Pfannkuche et al. tested attachment of ESCM on fibronectin and 18 additional surfaces. These analyses revealed that murine cardiomyocytes attached best on fibronectin-coated plates [43]. In the present study, we first wanted to determine, if the attachment capability to fibronectin differs among iPSC-CM of different developmental stages. The adhesion of iPSC-CM to fibronectin-coated dishes was numerically higher at day 14 than at day 16 or day 18 of differentiation, but the differences were not statistically significant.

Secondly, since fibronectin-coated plastic dishes do not reflect the complex in vivo ECM composition, attachment of iPSC-CM to decellularized ECM scaffolds was tested and supported the higher focal adhesion capability of day 14 iPCM. However, the number of attached cells was very low and even below the detection level in many samples, which hampered an exact quantification and may be one explanation for the loss of iPSC-CM after transplantation in recipient hearts. One limitation of this model and potential explanation for the low adhesion is that detergents used to decellularize the tissue may have impaired ECM compounds. SDS had a high efficiency in dsDNA removal, but also affected fiber structure of the ECM in a previous study [44], while Triton X-100 retained an intact fiber network, but left a higher amount of dsDNA fragments [45].

Fibronectin, Collagens, and Laminins interact with integrins on the surface of cardiomyocytes to mediate focal cell adhesion. In cardiomyocytes, some of the predominantly expressed integrin heterodimers are α1β1, α5β1, and α7β1, which are the main collagen, fibronectin, and laminin binding receptors [46]. Differences in integrin expression between day 14, day 16, and day 18 iPSC-CM were considered to be one reason for different adhesion capabilities in our experimental settings. However, gene array analysis of day 14, day 16, and day 18 iPSC-CM did neither reveal relevant differences in α1, α5, α7, β1, and β8 integrin subunit expression nor in integrin binding protein fibulin 1, fibroblast growth factor 1, and myosin heavy polypeptide 9 protein expression. Thus, the reason for the higher cell adhesion on ECM scaffolds, as well as the higher persistence of day 14 iPSC-CM after transplantation, remains unexplained. Potential explanations, which deserve further investigations in future studies, include differences in integrin expression on the protein level in day 14 vs. older iPSC-CM or morphological differences of the cells, which may influence cell adhesion capabilities. Moreover, stage-dependent vulnerability of iPSC-CM to the dissociation process may play a role. Lower rates of apoptosis or necrosis in day 14 iPSC-CM compared to day 16 and day 18 iPSC-CM could enhance the adhesion to extracellular matrix. However, due to the short periods of time used in the in vitro experiments from plating to wash-out of the cells, we consider that neither apoptosis nor necrosis play a major role in the higher adhesion of day 14 iPSC-CM.

A number of techniques have been applied to improve persistence of transplanted cells. Co-injection of biomaterials, which influence the microenvironment after cell transplantation and may close the injection channel, can improve cell retention. Encapsulation of cardiosphere-derived rat stem cells in hyaluronic acid-serum hydrogels led to a sixfold increased cell persistence after transplantation into non-infarcted rat myocardium [47]. Embedding human cardiac stem cells into a hydrogel matrix also increased long-term retention in mice threefold [48].

Labeling cells with magnetic particles allows cell guidance to an area of interest by applying a magnetic field. Vandergriff et al. demonstrated that magnetic labeling can increase persistence of cardiosphere-derived stem cells inside the infarcted myocardium fourfold [49].

Co-transplantation of mesenchymal stem cells and stem cell-derived cardiomyocytes seems to be highly efficient regarding the improvement of acute retention. The transplantation of in vitro grown microtissue composed of bone marrow-derived murine mesenchymal stem cells and murine ESCM resulted in a more than tenfold increase of cell retention in comparison to single cell suspensions of ESCM 1 day after transplantation [20]. However, there was a highly relevant cell loss at the following 2 days. Moreover, the mean persistence of single cells was very low even at day 1 after transplantation (0.6%, as compared to average retention of 5.7% at 6–7 days after transplantation of day 14 iPSC-CM in the present study).

In our study, iPSC-CM persistence was increased sixfold by using cells at an optimal developmental stage. In comparison to established techniques to improve cell persistence, which require considerable technical efforts, the choice of the optimal developmental stage appears to have a similar or even better effect on persistence and involves few technical efforts.

Besides persistence, the functional integration of transplanted cells is a prerequisite for an efficient cell replacement therapy. The maximal stimulation frequency without conduction blocks, which is a measure of the quality of electrical integration, was reported to be higher in intermediate stage fetal cardiomyocytes than in late stage fetal cardiomyocytes (the early stage could not be analyzed due to the low persistence) [26]. In the present study, the quality of electrical integration of day 14 iPSC-CM was similar and even numerically (but not significantly) higher than that of day 16 iPSC-CM, demonstrating that the improved persistence is not at the expense of functional integration.

The low number of analyzed preparations limited the evaluation of AP properties of iPSC-CM at different stages and host cardiomyocytes. Unlike iPSC-CM, fetal cardiomyocytes show a fast maturation after transplantation and possess adult-like AP properties after 12 days, if they are electrically integrated [21]. The maturation process of fetal cardiomyocytes is mainly characterized by a decrease in APD50 and an increase in APD90. The present study showed significant differences between host CM and transplanted day 14 and day 16 iPSC-CM 7 days after transplantation, in line with previous studies [22]. However, our AP measurements 7 days after transplantation revealed a significantly lower APD50 in day 16 as compared to day 14 iPSC-CM, which may be an indicator of a higher maturation stage of day 16 iPSC-CM.

The conduction properties of the myocardium depend on the geometry of the connected cells and number, size, and location of gap junction plaques between them [48]. In adult cardiomyocytes, the predominant distribution of gap junctions is end-to-end, located in intercalated discs. Immature cardiomyocytes, like iPSC-CM, show a more homogenous arrangement of connexins over the entire cell surface. After transplantation, the formation of gap junctions between the host and graft tissue is a prerequisitions for electrical integration of the transplanted iPSC-CM. The quality of electrical integration was assessed 7 days after transplantation in the present study. Due to the short period, optimal electrical integration was not expected, since one previous study showed an increasing the quality of electrical integration up to 6 to 8 months [22].

Cardiac gap junctions consist of connexins 40, 43, and 45. Gene array results revealed no significant changes in Gja1 (Connexin 43) and Gjc1 (Connexin 45) expression levels. Gja5 (Connexin 40) expression was only slightly lower in day 14 iPSC-CM (− 2.14 fold change between day 14 and day 16 iPSC-CM), which is consistent with our finding that the quality of electrical integration of day 14 and day 16 iPSC-CM is comparable 7 days after transplantation.

We used only one murine iPS cell line as a proof-of-principle for developmental stage-dependent persistence. The findings of our study might be cell line-specific and especially the exact stage, which offers the highest persistence after transplantation, might be different for different cell lines. In previous studies, we and other groups described substantial functional differences between different stem cell lines [50–52], so we consider it very likely that also persistence and adhesion will be influenced by the choice of cell line. Furthermore, non-murine cell lines will most probably behave differently, too, so translation into primate models and finally in clinical studies will require an additional investigation of stage-specific effects on persistence.

It is probable that the best stage for transplantation of iPSC-CM will also depend on cell culture and differentiation protocols. Moreover, as iPS cells started cardiac differentiation spontaneously in our experimental setting, not all cells may have attained the same stage of maturation at the same day of differentiation.

We used direct intramyocardial injection of iPSC-CM, which is one method of cell delivery that can be used in humans [53] and is easy to apply in the mouse model. It allows the visualization and precise targeting of the infarct and infarct border zone as well as the delivery of high numbers of cells. However, this technique itself may have an influence on cells persistence. Direct injection creates an injury of the host tissue that may trigger acute inflammatory responses and lead to lower cell survival [54]. Moreover, cell loss through the injection channel into the pericardial space [16] may especially occur in small animal models, where the distance between injection site and pericardial space and the length of the injection channel are very small. However, it is unlikely that this biased the differences in persistence of day 14 as compared to day 16 iPSC-CM, since cells at both developmental stages are similar in size and shape and will be subject to these limitations in a similar way.

We transplanted only day 14 and day 16 iPSC-CM, while we also used day 18 iPSC-CM for the in vitro studies. Earlier studies revealed a loss of cardiac commitment under prolonged culture in cardiac bodies [35]. Therefore, and since cell adhesion to ECM was below the detection level in day 18 iPSC-CM and a low persistence after transplantation was anticipated, we decided not to include day 18 iPSC-CM in the in vivo experiments.

## Conclusions

The present work demonstrates that the developmental stage at the time of transplantation is crucial for the persistence of transplanted murine iPSC-CM. This makes the choice of the optimal developmental stage an important step for a successful clinical translation of iPSC-CM-based therapies.

iPSC-CM at day 14 of differentiation showed the highest persistence after transplantation and the highest capability to adhere to ECM scaffolds in vitro; thus, better adhesion to host tissue and reduced wash out from the injection site may be mechanisms underlying the improved persistence of these cells as compared to more mature cells.

## Supplementary Information

**Additional file 1: Supplemental Fig. 1.** Gene array data for most common cardiac integrins and integrin binding proteins. All experiments were performed in triplicates made of different cell batches. *P*-values are shown in supplemental Table 2.

**Additional file 2: Supplemental Fig. 2.** Gene array data for gap junction protein α1 (connexin 43), α5 (connexin 40) and γ1 (connexin 45). All experiments were performed in triplicates made of different cell batches. P-values are shown in supplemental Table 4.

**Additional file 3.** Supplemental methods

**Additional file 4: Supplemental Table 1.** Gene array data for most common cardiac integrins and integrin binding proteins. **Supplemental Table 2.** Integrins and integrin binding proteins expression changes. **Supplemental Table 3.** Gene array data for most common cardiac gap junction proteins. **Supplemental Table 4.** Gap junction protein expression changes.

## Data Availability

The datasets used and/or analyzed during the current study are available from the corresponding author on reasonable request.

## Abbreviations

AP: Action potential
APD50: Action potential duration at 50% of repolarization
APD90: Action potential duration at 90% of repolarization
DMEM: Dulbecco’s modified Eagle’s medium
ECM: Extra cellular matrix
eGFP: Enhanced green fluorescent protein
ESC-CM: Embryonic stem cell-derived cardiomyocytes
Fbln1: Fibulin 1
FGF1: Fibroblast growth factor 1
Gja1: Gap junction protein α1
Gja5: Gap junction protein α5
Gjc1: Gap junction protein β1
IMDM: Iscove’s modified Dulbecco’s medium
IRES: Internal ribosomal entry site
Itga1: Integrin α1
Itag5: Integrin α5
Itga7: Integrin α7
Itgb1: Integrin β1
Itgb8: Integrin β8
MI: Myocardial infarction
MDP: Maximal diastolic potential
MSC: Mesenchymal stem cells
PAC: Puromycin *N*-acetyl-transferase
PBS^−/−^: Phosphate-buffered saline without calcium and magnesium
RIVA: Ramus interventricularis anterior
SDS: Sodium dodecyl sulfate
S.E.M.: Standard error of mean
Vmax: Upstroke velocity
