# Supplementary figures and images for: Persistence of intramyocardially transplanted murine induced pluripotent stem cell-derived cardiomyocytes from different developmental stages
Source: Stem Cell Res Ther. 2021 Jan 8;12:46. doi: 10.1186/s13287-020-02089-5 (PMC7792075; doi:10.1186/s13287-020-02089-5)

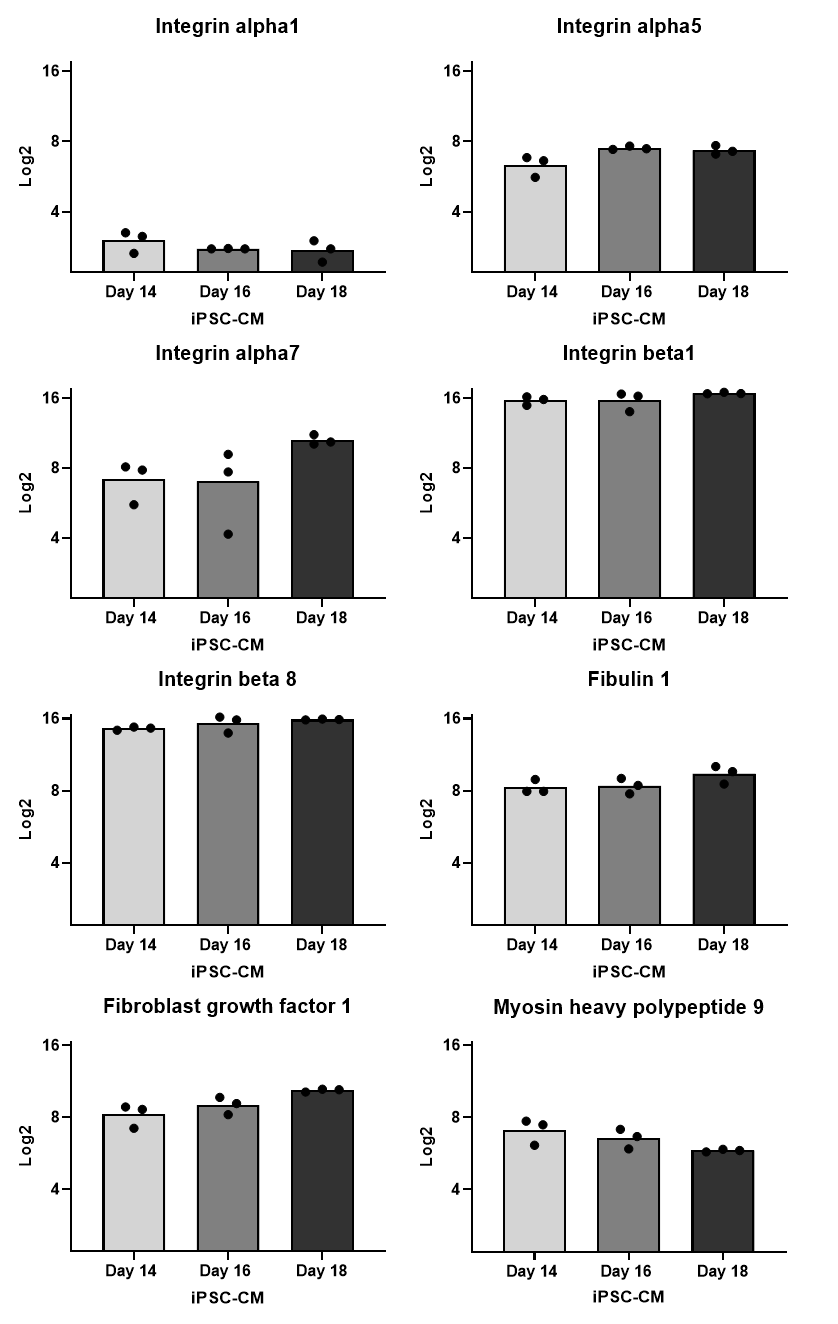

Supplement: Supplementary file 1 — Additional file 1: Supplemental Fig. 1. Gene array data for most common cardiac integrins and integrin binding proteins. All experiments were performed in triplicates made of different cell batches. P-values are shown in supplemental Table 2. [file 13287_2020_2089_MOESM1_ESM.tif]

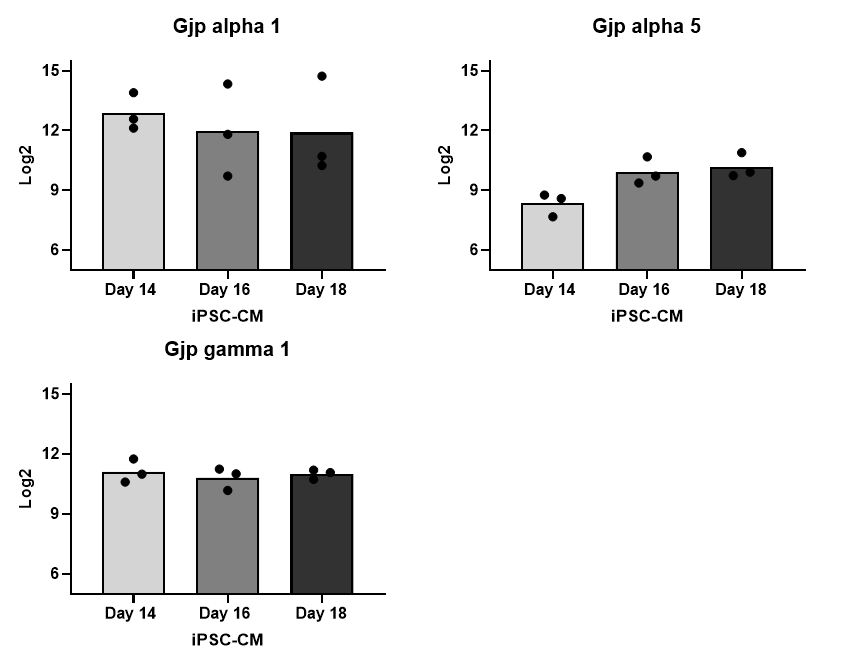

Supplement: Supplementary file 2 — Additional file 2: Supplemental Fig. 2. Gene array data for gap junction protein α1 (connexin 43), α5 (connexin 40) and γ1 (connexin 45). All experiments were performed in triplicates made of different cell batches. P-values are shown in supplemental Table 4. [file 13287_2020_2089_MOESM2_ESM.tif]
