# Supplementary material for: Persistence of intramyocardially transplanted murine induced pluripotent stem cell-derived cardiomyocytes from different developmental stages
Source: Stem Cell Res Ther. 2021 Jan 8;12:46. doi: 10.1186/s13287-020-02089-5 (PMC7792075; doi:10.1186/s13287-020-02089-5)
Supplement: Supplementary file 3 — Additional file 3. Supplemental methods [file 13287_2020_2089_MOESM3_ESM.docx]

**Supplemental methods**

*Microarray gene expression analysis*

To isolate total RNA for global gene expression analysis dissociated day 14, day 16 and day 18 iPSC-CM were collected. For each analysis, triplicates made of different cell batches were used. Microarray analysis was performed as described previously (1, 2). Briefly, total RNA was isolated using miRNeasy mini kit (Qiagen, Hilden, Germany), and quantified using NanoDrop (ND-1000, Thermo Fisher Scientific, Waltham, Massachusetts, USA). For microarray labelling, 100 ng total RNA isolated from iPSC-CM was used as a starting material, and after amplification 12.5 µg amplified RNA were hybridized on Mouse Genome 430 version 2.0 arrays (Affymetrix, Santa Clara, CA, USA) for 16 hrs at 45 °C. The arrays were washed and stained in Affymetrix Fluidics Station-450 according to the manufacturer’s instructions. For transcriptome analysis, 5.5 µg fragmented biotin-labeled ds cDNA were hybridized to Clariom™ S arrays (Clariom™ S arrays, Mouse, Applied Biosystems, Thermo Fisher Scientific). After staining, arrays were scanned with Affymetrix Gene-Chip Scanner-3000-7G, while quality control matrices were confirmed with Affymetrix GCOS software. Statistical data analysis has been performed using the Transcriptome Analysis Console (TAC) software from Thermo Fisher Scientific/Affymetrix after uploading of the .Cel files and analyzing the differentially expressed genes of day 14, day 16 and day 18 iPSC-CM. The probe set intensity values were generated by “Signal Space Transformation” (SST)-RMA normalization method, background correction, quantile normalization and log2 transformation. Comparison was performed by the ANOVA method (ebayes) to generate the differentially regulated transcripts with at least a 2-fold change (p-value < 0.05).

1. Meganathan K, Jagtap S, Wagh V, Winkler J, Gaspar JA, Hildebrand D, et al. Identification of thalidomide-specific transcriptomics and proteomics signatures during differentiation of human embryonic stem cells. PLoS One. 2012;7(8):e44228.

2. Heras-Bautista CO, Katsen-Globa A, Schloerer NE, Dieluweit S, Abd El Aziz OM, Peinkofer G, et al. The influence of physiological matrix conditions on permanent culture of induced pluripotent stem cell-derived cardiomyocytes. Biomaterials. 2014;35(26):7374-85.
