# Supplementary material for: Persistence of intramyocardially transplanted murine induced pluripotent stem cell-derived cardiomyocytes from different developmental stages
Source: Stem Cell Res Ther. 2021 Jan 8;12:46. doi: 10.1186/s13287-020-02089-5 (PMC7792075; doi:10.1186/s13287-020-02089-5)

**Supplemental tables**

**Supplemental table 1:** Gene array data for most common cardiac integrins and integrin binding

proteins.


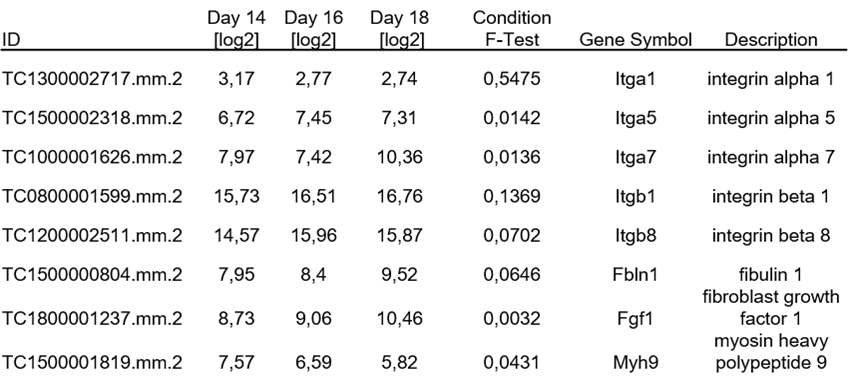


**Supplemental table 2:** Integrins and integrin binding proteins expression changes.


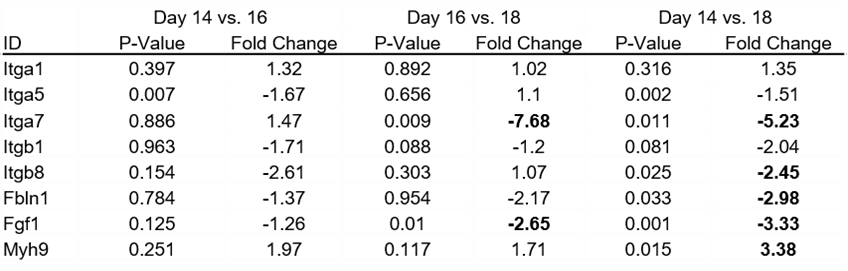


**Supplemental table 3:** Gene array data for most common cardiac gap junction proteins.


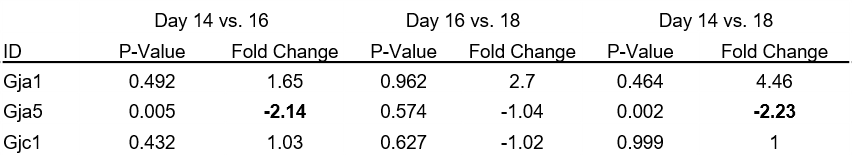

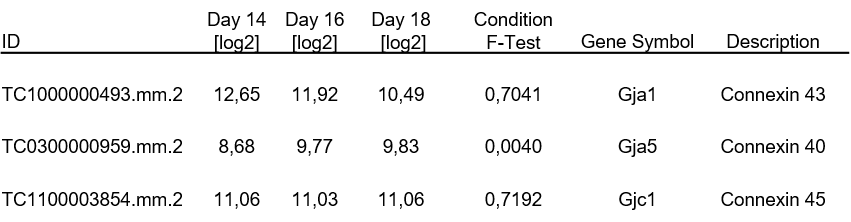

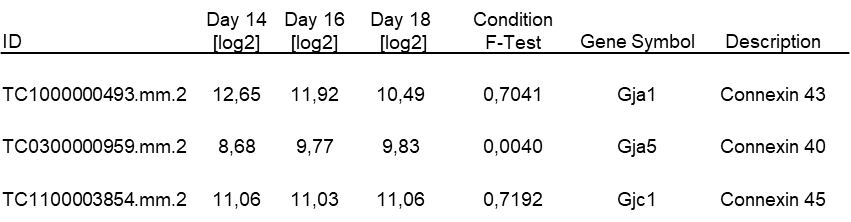


**Supplemental table 4: P**-values and fold change for gap junction protein expression changes.

**Supplemental table 4:** Gap junction protein expression changes.
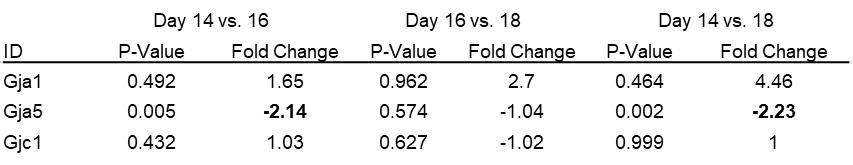

Supplement: Supplementary file 4 — Additional file 4: Supplemental Table 1. Gene array data for most common cardiac integrins and integrin binding proteins. Supplemental Table 2. Integrins and integrin binding proteins expression changes. Supplemental Table 3. Gene array data for most common cardiac gap junction proteins. Supplemental Table 4. Gap junction protein expression changes. [file 13287_2020_2089_MOESM4_ESM.docx]
